# Supplementary material for: Ssd1 and Gcn2 suppress global translation efficiency in replicatively aged yeast while their activation extends lifespan
Source: eLife. 2018 Aug 17;7:e35551. doi: 10.7554/eLife.35551 (PMC6097839; doi:10.7554/eLife.35551)
Supplement: Supplemental file 2. — Ribo-seq data during aging. The complete list of sorted genes including their gene expression values and translation efficiency calculations. [file elife-35551-fig2.docx]

**Table S2.** Yeast strains used in this study

| **Strain** | **Genotype** | **Reference** |
| --- | --- | --- |
| ZHY2 | *MATα ade2::hisG his3 leu2 met15Δ::ADE2 trp1Δ63 ura3Δ0::URA3 hoΔ::SCW11pr-Cre-EBD78-NatMX loxP-UBC9-loxP-LEU2 loxP-HPMX* | *(Hu et al., 2014)* |
| ZHY3 | *BY4741 MAT*a *his3Δ1 leu2Δ0 met15Δ0 ura3Δ0 GAL1-SSD1* | *This study* |
| ZHY6 | *MATα ade2::hisG his3 leu2 met15Δ::ADE2 trp1Δ63 ura3Δ0::URA3 hoΔ::SCW11pr-Cre-EBD78-NatMX loxP-UBC9-loxP-LEU2 loxP-HPMX SSD1::HA* | *This study* |
| ZHY7 | *MATα ade2::hisG his3 leu2 met15Δ::ADE2 trp1Δ63 ura3Δ0::URA3 hoΔ::SCW11pr-Cre-EBD78-NatMX loxP-UBC9-loxP-LEU2 loxP-HPMX ssd1Δ::KanMX6* | *This study* |
| ZHY8 | *MATα ade2::hisG his3 leu2 met15Δ::ADE2 trp1Δ63 ura3Δ0::URA3 hoΔ::SCW11pr-Cre-EBD78-NatMX loxP-UBC9-loxP-LEU2 loxP-HPMX gcn2Δ::KanMX6* | *This Study* |
| BY4741 | *MAT*a *his3Δ1 leu2Δ0 met15Δ0 ura3Δ0* |  |
| BY4742 | *MATalpha his3Δ1 leu2Δ0 lys2Δ0 ura3Δ0* |  |
| BY4742  *gcn2∆* | *MATalpha his3Δ1 leu2Δ0 lys2Δ0 ura3Δ0 gcn2∆::KanMX* |  |
| BY4742  *gcn4∆* | *MATalpha his3Δ1 leu2Δ0 lys2Δ0 ura3Δ0 gcn4∆::KanMX* |  |
| H1402 | *MAT*a *leu2-3 leu2-112ura3-52ino1GCN2 (His4-lacZ, ura3-52)* | *(Hannig, Williams, Wek, & Hinnebusch, 1990)* |
| F113 | *MAT*a *ura3-52 ino1 can1* | *(Abastado, Miller, Jackson, & Hinnebusch, 1991)* |
| ZHY9 | *MATα ade2::hisG his3 leu2 met15Δ::ADE2 trp1Δ63 ura3Δ0::URA3 hoΔ::SCW11pr-Cre-EBD78-NatMX loxP-UBC9-loxP-LEU2 loxP-HPMX LSM1::GFP* | *This study* |
| ZHY17 | *MAT*a  *ino1 can1 ura3-52::URA3* | *This study* |
| ZHY12 | *MAT*a *ura3-52 ino1 can1* GCN4^c^ inserted at the URA3 locus | *This study* |
| ZHY18 | *MAT*a *ura3-52 ino1 can1 GFP-ATG8::URA3* GCN4^c^ inserted at the URA3 locus | *This study* |
| ZHY19 | *MAT*a *ura3-52 ino1 can1 GFP-ATG8::URA3* | *This study* |
| ZHY20 | *MATa ino1 can1 ura3-52::URA3 atg1Δ:KanMX6* | *This study* |
| ZSY2112 | *MATa ino1 can1 ura3-52::URA3 atg8Δ:KanMX6* | *This study* |
| ZSY2111 | *MATa ura3-52 ino1 can1* GCN4^c^*inserted at the*URA3 *locus atg8Δ:KanMX6* | *This study* |
| ZHY16 | *MATa ura3-52 ino1 can1* GCN4^c^*inserted at the*URA3 *locus atg1Δ:KanMX6* | *This study* |
| ZHY22 | *MAT alpha ade2::HisG his3 leu2 met15∆::ADE2 trp1∆63 ho∆::SCW11pr-CRE-EBD78-NatMX loxP-UBC9-loxP-LEU2 loxP-CDC20-Intron-loxP-HPHMX ssd1::TRP1 gcn2::KanMX6* | *This study* |
| WLY176 | *SEY6210 pho13∆ pho8∆60::HIS3* | *(Kanki et al., 2009)* |
| ZSY2119 | *WLY176* GCN4^c^*inserted at the*URA3 *locus* | *This study* |
| WLY192 | *SEY6210 pho13∆KAN pho8∆60::URA3 atg1∆::HIS5* | *(Kanki et al., 2009)* |
| ZSY2113 | *WLY176* GCN4^c^*inserted at the*URA3 *locus atg1∆::HPH* | *This study* |
| ZSY2117 | *WLY176 atg8∆::HPH* | *This study* |
| ZSY2118 | *WLY176* GCN4^c^*inserted at the*URA3 *locus atg8∆::HPH* | *This study* |

**Table S3. Plasmids used in this study**

| **Plasmid** | **Description** | **Reference** |
| --- | --- | --- |
| C1683 | pRS423-*IMT4* | (Roll-Mecak, Alone, Cao, Dever, & Burley, 2004). |
| B139 | pRS306-GCN4c uORFs deleted | (Hinnebusch, 1985). |
| pRS306 | Integrative empty vector with *URA3* selection | ATCC |
| pRS423 | 2 micro empty vector with *HIS3* selection | ATCC |
| pVW31 | GCN4-firefly luciferase with *URA3* selection | (Steffen et al., 2008). |

Abastado, J. P., Miller, P. F., Jackson, B. M., & Hinnebusch, A. G. (1991). Suppression of ribosomal reinitiation at upstream open reading frames in amino acid-starved cells forms the basis for GCN4 translational control. *Mol Cell Biol, 11*(1), 486-496. Retrieved from <https://www.ncbi.nlm.nih.gov/pubmed/1986242>

Hannig, E. M., Williams, N. P., Wek, R. C., & Hinnebusch, A. G. (1990). The translational activator GCN3 functions downstream from GCN1 and GCN2 in the regulatory pathway that couples GCN4 expression to amino acid availability in Saccharomyces cerevisiae. *Genetics, 126*(3), 549-562. Retrieved from <https://www.ncbi.nlm.nih.gov/pubmed/2249755>

Hinnebusch, A. G. (1985). A hierarchy of trans-acting factors modulates translation of an activator of amino acid biosynthetic genes in Saccharomyces cerevisiae. *Mol Cell Biol, 5*(9), 2349-2360. Retrieved from <https://www.ncbi.nlm.nih.gov/pubmed/3915540>

Hu, Z., Chen, K., Xia, Z., Chavez, M., Pal, S., Seol, J. H., . . . Tyler, J. K. (2014). Nucleosome loss leads to global transcriptional up-regulation and genomic instability during yeast aging. *Genes Dev, 28*(4), 396-408. doi:10.1101/gad.233221.113

Kanki, T., Wang, K., Baba, M., Bartholomew, C. R., Lynch-Day, M. A., Du, Z., . . . Klionsky, D. J. (2009). A genomic screen for yeast mutants defective in selective mitochondria autophagy. *Mol Biol Cell, 20*(22), 4730-4738. doi:10.1091/mbc.E09-03-0225

Roll-Mecak, A., Alone, P., Cao, C., Dever, T. E., & Burley, S. K. (2004). X-ray structure of translation initiation factor eIF2gamma: implications for tRNA and eIF2alpha binding. *J Biol Chem, 279*(11), 10634-10642. doi:10.1074/jbc.M310418200

Steffen, K. K., MacKay, V. L., Kerr, E. O., Tsuchiya, M., Hu, D., Fox, L. A., . . . Kaeberlein, M. (2008). Yeast life span extension by depletion of 60s ribosomal subunits is mediated by Gcn4. *Cell, 133*(2), 292-302. doi:S0092-8674(08)00288-2 [pii]

10.1016/j.cell.2008.02.037
